# Optimization of dosing regimens for the long-acting growth hormone pegpesen: A population PK/PD modeling approach

**DOI:** 10.1007/s40618-025-02749-4

**Published:** 2025-11-05

**Authors:** Youni Zhao, Fenfang Zou, Jianbo Gu, Ruoyi He, Yalin Yin

**Affiliations:** Xiamen Amoytop Biotech Co., Ltd, Xiamen, Fujian P. R. China

**Keywords:** Y-shape pegylated RhGH, Growth hormone, Growth hormone deficiency, Long-acting growth hormone

## Abstract

**Purpose:**

Long-acting growth hormone (LAGH) provides a convenient treatment for children with growth hormone deficiency (GHD), but challenges such as waning growth velocity (GV) and dosing inflexibility persist. This study aimed to explore optimized dosing strategies for Pegpesen, a novel LAGH, using population pharmacokinetic/pharmacodynamic (PopPK/PD) modeling to improve therapeutic outcomes.

**Methods:**

A PopPK/PD model was developed using data from Phase 1–3 trials of Pegpesen. Two strategies were simulated in 292 GHD patients: (1) Dose up-titration, starting at 0.14 mg/kg/week and increasing by 12.3%, 18.9%, and 26.0% every 3 months to a maximum of 0.28 mg/kg/week. (2) Weight-banded dosing, evaluating the suitability of fixed doses for children within ± 1.78 kg and ± 3.57 kg of a target weight (fixed dose/0.14 kg). Primary evaluation metrics included 12- and 24-month GV, IGF-1 levels, and PK/PD profiles.

**Results:**

The up-titration strategy dose-dependently increased 12-month GV (9.51–9.88 cm/year), which then converged by 24 months, suggesting that saturation was reached before the second year of treatment. IGF-1 levels remained within safe range. For weight-banded dosing, PK/PD profiles for subjects within ± 1.78 kg of the target weight were comparable to standard weight-based dosing, whereas profiles for the ± 3.57 kg range showed significant divergence.

**Conclusion:**

This PopPK/PD model for LAGH Pegpesen in GHD children proposes a dose up-titration regimen that effectively counteracts declining GV while maintaining safety, and a simplified weight-banded dosing system that enhances treatment convenience without compromising efficacy.

**Supplementary Information:**

The online version contains supplementary material available at 10.1007/s40618-025-02749-4.

## Introduction

Since 1985, recombinant human growth hormone (rhGH) replacement therapy has been the standard of care for growth hormone deficiency (GHD) [1]. However, outcomes with daily rhGH are often suboptimal, as many children with GHD fail to reach the average height of their age-, sex-, and ethnicity-matched peers [2–5]. This suboptimal outcome stems from various factors, with poor adherence to the daily injection regimen being a major contributor [6, 7]. Multiple studies have reported a significant proportion of missed injections (approximately 30%) among patients on daily rhGH [8]. A real-world study in Spain demonstrated that each 10% increase in adherence among children with GHD was associated with a 1.1 cm/year increase in growth velocity (GV) [9]. Similarly, a real-world study in the United States highlighted the importance of high adherence for achieving greater adult height in children with GHD (163.0 cm vs. 156.9 cm) [10]. Consequently, since 1999, various long-acting growth hormone (LAGH) formulations, developed using different technologies to extend the rhGH half-life, have been marketed. These agents allow for once-weekly administration, aiming to reduce treatment burden, improve adherence, and ultimately enhance linear growth outcomes [11, 12]. To date, three LAGH products have been approved by the FDA and EMA [13–15], two in China [16, 17], and one in South Korea [18].

However, several challenges associated with current LAGH therapies remain unresolved. First, LAGH products currently approved for pediatric GHD are administered at a fixed weight-based dose. For example, the recommended dosages are 0.16 mg/kg/week for somapacitan, 0.24 mg/kg/week for lonapegsomatropin, and 0.66 mg/kg/week for somatrogon [13–15]. In contrast, GHD guidelines recommend that the dosage of daily rhGH be individualized based on various factors, such as the peak GH level in stimulation tests, the presence of concomitant hormone deficiencies, pubertal status, degree of epiphyseal maturation, and the patient’s response to therapy, with dosages typically ranging from 0.02 mg/kg/day to 0.05 mg/kg/day [1, 19, 20]. Second, studies have found that GV tends to wane over time in children treated with either daily rhGH or LAGH [21–25]. A retrospective cohort study of prepubertal children with short stature secondary to GHD or being born small for gestational age (SGA) on daily rhGH found that the mean GV for the GHD and SGA groups over years 1–3 were 9.6 and 8.8, 7.8 and 6.7, and 7.2 and 6.3 cm/year, respectively [21]. This trend is also well-documented for LAGH. For instance, a four-year study of the LAGH somapacitan, administered at a constant dose, reported a progressive decline in mean GV from 11.5 cm/year in the first year to 7.4 cm/year by the fourth [24]. This phenomenon does not fully meet the desire of patients and their families for sustained catch-up growth. Since numerous studies confirm a positive dose-response relationship for GH [26–28], dose up-titration presents a potential strategy to counteract this decline. This approach could maintain the high GV seen early in treatment, thereby improving overall efficacy, yet it has not been fully explored for LAGH.

Another challenge is the current practice of weight-based dosing recommended by guidelines for both daily rhGH and LAGH [1, 19, 20]. As children grow, their weight constantly changes, which can necessitate frequent adjustments to dosage and product strength, adding to the treatment burden. A weight-banded dosing model, where a single product strength can be used for children within a certain weight range, could simplify treatment. However, to our knowledge, such an approach has not yet been explored for any LAGH.

Pegpesen (Xiamen Amoytop Biotech Co., Ltd), a recently marketed LAGH in China, is a 40 kDa Y-shaped polyethylene glycol (PEG)-modified rhGH. Due to the covalent conjugation of the Y-shaped PEG to a specific, high-activity site on the rhGH molecule, Pegpesen can be initiated at a relatively low starting dose in children with GHD [17, 29]. A Phase 3 study confirmed that once-weekly Pegpesen at 0.14 mg/kg/week achieved comparable GV to daily rhGH at 0.035 mg/kg/day, with a similar safety profile [17]. Pegpesen has also completed a Phase 2 basket trial (NCT05838885) in children with ISS, SGA, or Turner Syndrome (TS), in which once-weekly Pegpesen at 0.28 mg/kg/week demonstrated comparable GV and safety to daily rhGH at 0.067 mg/kg/day (data not yet published). These studies establish a dose-effect relationship for Pegpesen within the 0.14–0.28 mg/kg/week range.

To address the aforementioned therapeutic challenges of LAGH, this study utilized data from the Phase 1, 2, and 3 clinical trials of Pegpesen to develop a population pharmacokinetic/pharmacodynamic (PopPK/PD) model. Using this model, we simulated the efficacy and safety of two alternative dosing strategies for Pegpesen in children with GHD over a 24-month period: first, a dose up-titration regimen starting at 0.14 mg/kg/week with periodic increases (up to a maximum of 0.28 mg/kg/week); and second, a weight-banded dosing regimen where children within a specific weight range receive the same product strength.

## Methods

### Data sources and modeling software

Population modeling was performed using NONMEM (non-linear mixed-effects model, v7.5.0; ICON Development Solutions, Ellicott City, MD, USA), with Perl-speaks-NONMEM (PsN, v4.8.1; Uppsala University, Uppsala, Sweden) as a run-management tool. R (v4.1.3; R Core Team [2022], R Foundation for Statistical Computing, Vienna, Austria) was used for exploratory data analysis, data management, and visualization of modeling results. The first-order conditional estimation with interaction (FOCEI) method was used for parameter estimation in the PK/PD models. The PK/PD models were constructed using the ADVAN subroutines within the PREDPP library of NONMEM.

Data from a Phase 1 study of Pegpesen in healthy adult subjects (NCT01339182) and a Phase 2/3 study in children with GHD (NCT04513171) were used to develop the PopPK model for Pegpesen. A sequential modeling approach was then applied, integrating the final PopPK model with PD data from the studies to establish the final PopPK/PD model. Simulations of different dosing regimens were then performed based on the 292 individual patients with GHD from the Phase 2/3 study.

### Trials providing data

The Phase 1 study of Pegpesen (NCT01339182) was a single-center, randomized, open-label, single-ascending-dose, active-controlled Phase 1 trial evaluating the tolerability, PK, and PD characteristics of Pegpesen in healthy male subjects. A total of 36 healthy male subjects were enrolled and received dosing in two stages. In stage one, subjects received subcutaneous injections of the active comparator (Saizen^®^, Serono, Switzerland) once daily for 7 consecutive days, followed by a 14-day washout period. In stage two, subjects received a single dose of Pegpesen. Subjects were grouped according to the Pegpesen dose received. Each subject was randomized to receive either 0.03 mg/kg/day or 0.05 mg/kg/day of Saizen^®^ in stage one, followed by a single subcutaneous injection of Pegpesen at one of five dose levels in stage two: 0.01 mg/kg/week, 0.03 mg/kg/week, 0.06 mg/kg/week, 0.12 mg/kg/week, or 0.2 mg/kg/week (Table S1).

The Phase 2/3 study (NCT04513171) was a multicenter, randomized, open-label, active-controlled, combined Phase 2/3 clinical trial evaluating Pegpesen for the treatment of children with GHD. The trial consisted of two stages. Stage one enrolled 43 subjects, primarily for optimal dose-finding. Stage two enrolled 391 subjects for efficacy and safety confirmation. Detailed information on the study design, inclusion and exclusion criteria, statistical methods, analytical assays, and baseline characteristics of the subjects has been previously published [17, 29].

The study protocol for each trial was approved by the Institutional Review Board/Ethics Committee at all participating sites. Lead site approvals were granted for the Phase 1 trial (Approval No. 2010L01901) and the combined Phase 2/3 trial (Approval No. [2018] Lunshenzi (261)-5). All trials were conducted in accordance with the International Council on Harmonization guidelines on Good Clinical Practice, the ethical principles of the Declaration of Helsinki, and Chinese regulatory requirements. Written informed consent was obtained from the subjects, parents, and/or legal guardians of the children, and assent was obtained from children as appropriate for their age before study enrollment.

### Dose up-titration simulation

An indirect response (IDR) model, describing the stimulation of insulin-like growth factor-1 (IGF-1) production by Pegpesen, was used to characterize the dynamics of IGF-1 following Pegpesen administration. A direct effect model was constructed to describe the dynamics of GV over time, using the area under the curve (AUC) during the dosing interval preceding each GV measurement as the exposure parameter.

Starting with an initial regimen of Pegpesen 0.14 mg/kg once weekly (QW), subsequent dosing regimens involving dose up-titration every 3 months at rates of 12.3%, 18.9%, or 26.0% were simulated. These rates were designed to reach the target dose of 0.28 mg/kg/week within 18, 12, or 9 months, respectively. The time course of GV over a 24-month treatment period was simulated for each regimen to observe the changes in growth effects resulting from the dose adjustments (Table 1). To assess the safety profile of the different up-titration levels, the time course of IGF-1 standard deviation score (SDS) during the week preceding each dose administration every 3 months was simulated based on the IGF-1 PK/PD model.

**Table 1:** Dose escalation regimen

Time (month)	Corresponding dose (mg/kg/week)		
	Dose increase by 12.3%	Dose increase by 18.9%	Dose increase by 26.0%
0	0.14	0.14	0.14
3	0.16	0.17	0.18
6	0.18	0.20	0.22
9	0.20	0.24	0.28
12	0.22	0.28	0.28
15	0.25	0.28	0.28
18	0.28	0.28	0.28
21	0.28	0.28	0.28
24	0.28	0.28	0.28

The therapeutic dose range for Pegpesen (0.14–0.28 mg/kg/week) was established through pharmacometric modeling and subsequently verified in multiple clinical trials. Model predictions indicated that the lower dose (0.14 mg/kg/week) mirrors the effects of standard daily rhGH (0.035 mg/kg/day), whereas the higher dose (0.28 mg/kg/week) corresponds to high-dose daily rhGH (0.067 mg/kg/day). The validity of this dose-response profile was confirmed in Phase 3 studies of GHD [17]. Furthermore, dose-equivalence simulations confirmed that Pegpesen 0.14–0.28 mg/kg/week and daily rhGH 0.035–0.067 mg/kg/day (0.1–0.2 IU/kg/day) yield comparable IGF-1responses at their respective dose levels (Table S2). Collectively, these findings validate the 0.14–0.28 mg/kg/week range as a safe and effective therapeutic window for Pegpesen, demonstrating a well-defined dose-dependent effect on both growth promotion and IGF-1 modulation.

### Weight-banded dosing simulation

To facilitate clinical application, seven fixed-dose groups were established based on the primary product strengths of Pegpesen (2, 2.5, 3, 3.5, 4, 4.5, and 5 mg) to evaluate suitable fixed-dose regimens for subjects with different body weights. The reference regimen was Pegpesen 0.14 mg/kg/week (the regimen used in the Phase 3 trial). The corresponding body weights for each fixed dose were 14.29, 17.86, 21.43, 25.00, 28.57, 32.14, and 35.71 kg, respectively. Since the fixed doses increased in 0.5 mg increments, the body weight range for each fixed-dose group was defined not to exceed the target weight ± 3.57 kg (calculated as 0.5 mg / 0.14 mg/kg). Consequently, IGF-1 time profiles were predicted for subjects with body weights of fixed dose/0.14 kg, fixed dose/0.14 ± 3.57 kg, and fixed dose/0.14 ± 1.78 kg (i.e., 3.57/2 kg). The ± 1.78 kg range was added as an additional simulation scenario due to concerns that a ± 3.57 kg weight range might be too wide, and halving this range could provide a more conservative estimate (Table 2). Given the significant differences in weight and height distributions among children of different ages and sexes, a virtual cohort of 70 pediatric GHD patients was generated. Based on height and weight data for Chinese children from the literature [30], age and height information for male and female subjects whose median weight most closely matched the target weights of the seven groups was obtained. This resulted in 70 virtual patients (10 subjects per fixed-dose group, with varying weights and a 1:1 male-to-female ratio). Using the final PopPK and PK/PD models, 500 simulations were performed to predict the PK, IGF-1, IGF-1 SDS, and GV characteristics of these virtual subjects after multiple administrations of the corresponding fixed doses.

**Table 2:** Weight-based segmented dosing regimen

Fixed dose (mg)	Simulated body weights (kg)				
	Target weight (kg)^[a]^	Target weight − 3.57 (kg)^[b]^	Target weight − 1.78 (kg)^[b]^	Target weight + 1.78 (kg)^[b]^	Target weight + 3.57 (kg)^[b]^
2	14.29	10.72	12.51	16.07	17.86
2.5	17.86	14.29	16.08	19.64	21.43
3	21.43	17.86	19.65	23.21	25.00
3.5	25.00	21.43	23.22	26.78	28.57
4	28.57	25.00	26.79	30.35	32.14
4.5	32.14	28.57	30.36	33.92	35.71
5	35.71	32.14	33.93	37.49	39.28

^[a]^The Target Weight is the body weight corresponding to the reference regimen of 0.14 mg/kg/week (i.e., Target Weight = Fixed Dose /0.14)

^[b]^The other Simulated Body Weights at Range Boundaries represent the lower and upper limits of a wide (± 3.57 kg) and narrow (± 1.78 kg) weight range explored in the simulation

### Sampling schedules

In the Phase 1 trial (NCT01339182), PK sampling times were: 30 min before the first dose (0 h) and at 6, 12, 15, 24, 48, 72, 96, 120, 144, 168, 192, 240, 288, and 336 h post-dose. The PK/PD sampling schedules for the Phase 2 and 3 trials (NCT04513171) are described in recently published articles [17, 29].

## Results

### Data for analyses

PopPK model: A total of 2655 PK observations from 36 healthy subjects in the Phase 1 trial and from 292 subjects (*n* = 31 in Phase 2, *n* = 261 in Phase 3) treated with Pegpesen in the Phase 2/3 trial were included in the Pegpesen PopPK analysis.

PopPK/PD model: A total of 2466 IGF-1 observations and 2188 GV observations from the 292 subjects treated with Pegpesen in the Phase 2/3 trial were used for the Pegpesen PopPK/PD analysis.

### Simulation results for dose up-titration regimens

The 12-month annualized GV results demonstrated that as the dose escalation rate increased, the GV achieved with Pegpesen also showed a corresponding rise. Specifically, the annualized GVs for the 12.3%, 18.9%, and 26.0% up-titration groups were 9.51, 9.85, and 9.88 cm/year, respectively, indicating a positive dose-response relationship for the growth-promoting effect of Pegpesen. These three groups reached the target dose of 0.28 mg/kg/week at 18, 12, and 9 months, respectively, and then continued treatment at this dose until 24 months. At the 24-month time point, the annualized GV for all groups was 9.35 cm/year, suggesting that a saturation effect of the dose increase had been reached (Table 3).

**Table 3:** Growth velocity and IGF-1 SDS in different dose escalation groups

	Growth velocity (cm/year)			Proportion of IGF-1 SDS > + 2			Mean IGF-1 SDS		
	Baseline	12th month	24th month	Baseline	12th month	24th month	Baseline	12th month	24th month
Dose increase by 12.3%	3.57 (1.06)	9.51 (2.35)	9.35 (2.16)	0	4.5%	10.6%	-1.27 (0.55)	0.37 (0.75)	0.82 (0.78)
Dose increase by 18.9%	3.57 (1.06)	9.85 (2.34)	9.35 (2.16)	0	7.5%	10.6%	-1.27 (0.55)	0.67 (0.75)	0.82 (0.78)
Dose increase by 26.0%	3.57 (1.06)	9.88 (2.35)	9.35 (2.16)	0	8.6%	10.6%	-1.27 (0.55)	0.73 (0.76)	0.82 (0.78)

Data are presented as mean (SD) for Growth Velocity and Mean IGF-1 SDS. Abbreviations: IGF-1, insulin-like growth factor-1; SDS, standard deviation score

A similar trend was observed for IGF-1 SDS. At 12 months of treatment, the proportions of patients with IGF-1 SDS exceeding + 2 were 4.5%, 7.5%, and 8.6% in the 12.3%, 18.9%, and 26.0% up-titration groups, respectively. By 24 months, the proportion of patients with IGF-1 SDS > + 2 was 10.6% in all groups, again indicating a dose saturation effect (Table 3). Overall, IGF-1 levels remained within a safe and manageable range under the simulated up-titration regimens.

### Simulation results for weight-banded dosing

Among the seven fixed-dose groups (ranging from 2 to 5 mg in 0.5 mg increments), the fixed dose/0.14 ± 3.57 kg group showed substantial differences in PK exposure compared to the other weight groups (fixed dose/0.14 and fixed dose/0.14 ± 1.78 kg) (Fig. S1).

**Fig. 1:**
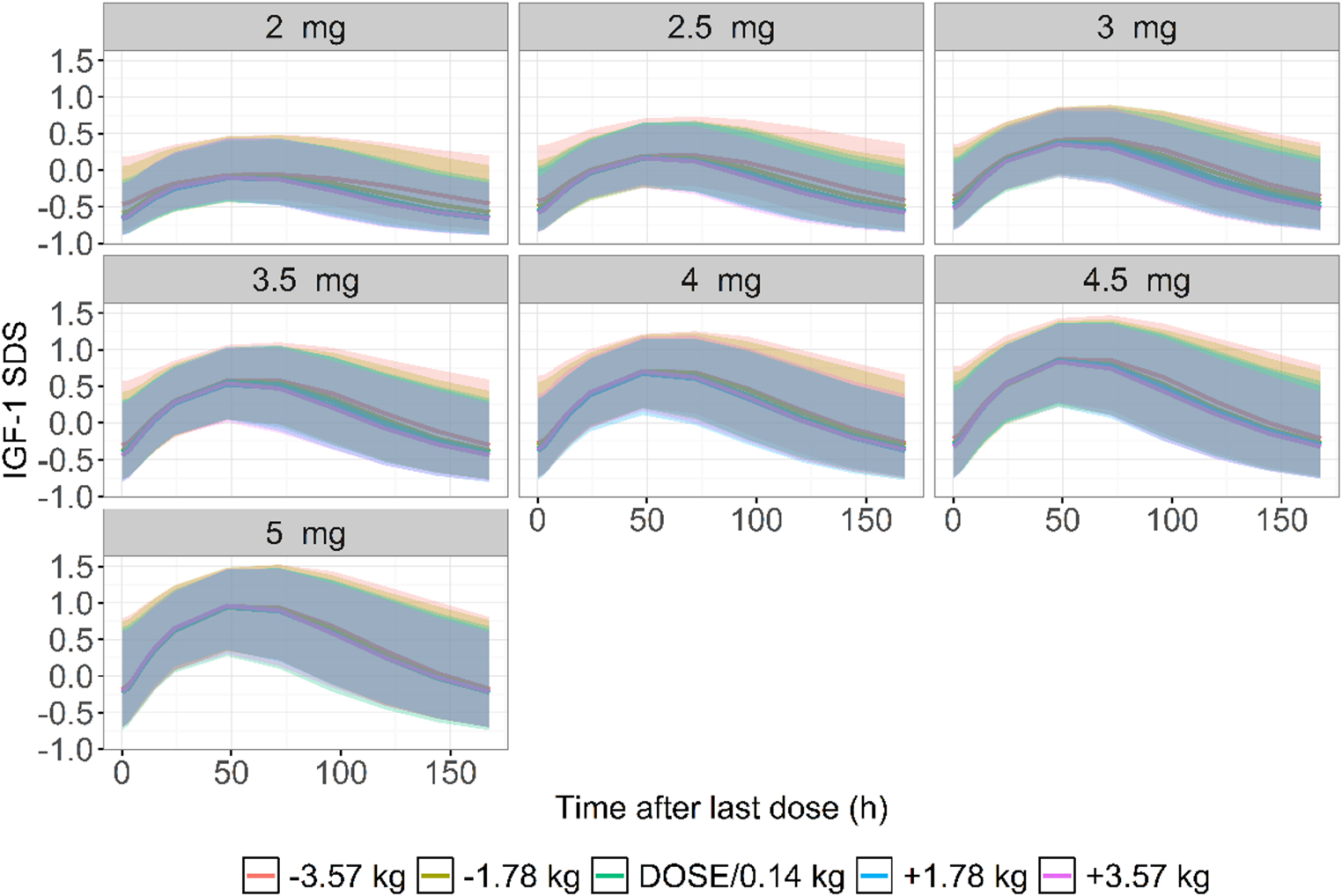
Time-course of steady-state IGF-1 SDS by fixed-dose stratified weight groups. Abbreviations: IGF-1, insulin-like growth factor-1; SDS, standard deviation score

When the baseline IGF-1 SDS for all subjects was standardized to -1, simulation of IGF-1 SDS at steady state (Week 12) [17, 29], showed that the 90% prediction intervals for the fixed dose/0.14 and fixed dose/0.14 ± 1.78 kg groups largely overlapped. In contrast, the 90% prediction interval for the fixed dose/0.14 ± 3.57 kg group slightly extended beyond that of the fixed dose/0.14 group (Fig. 1). Furthermore, simulation of GV levels at Week 12 revealed that the fixed dose/0.14 group and the fixed dose/0.14 ± 1.78 kg group had similar GV outcomes at their corresponding fixed doses. The difference in simulated GV between these two groups tended to decrease as the fixed dose increased (Table 4).

**Table 4:** Growth velocity under different weight-based segmentation regimens

Fixed dose, mg	Weight-band regimen, kg	GV, Mean (SD), cm/year	GV difference compared with the fixed dose/0.14 group, cm/year
2	10.72 (14.29–3.57)	14.3 (4.55)	0.6
	12.51 (14.29–1.78)	14.1 (4.51)	0.4
	**14.29 (2/0.14)**	**13.7 (4.51)**	**0.0**
	16.07 (14.29 + 1.78)	13.2 (4.40)	-0.5
	17.86 (14.29 + 3.57)	13.0 (4.54)	-0.7
2.5	14.29 (17.86–3.57)	12.4 (3.86)	0.5
	16.08 (17.86–1.78)	12.2 (3.75)	0.3
	**17.86 (2.5/0.14)**	**11.9 (4.03)**	**0.0**
	19.64 (17.86 + 1.78)	11.6 (3.94)	-0.3
	21.43 (17.86 + 3.57)	11.2 (3.74)	-0.7
3	17.86 (21.43–3.57)	11.3 (3.64)	0.4
	19.65 (21.43–1.78)	11.1 (3.5)	0.2
	**21.43 (3/0.14)**	**10.9 (3.58)**	**0.0**
	23.21 (21.43 + 1.78)	10.7 (3.49)	-0.2
	25.00 (21.43 + 3.57)	10.6 (3.48)	-0.3
3.5	21.43 (25.00-3.57)	10.5 (3.16)	0.3
	23.22 (25.00-1.78)	10.4 (3.32)	0.2
	**25.00 (3.5/0.14)**	**10.2 (3.29)**	**0.0**
	26.78 (25.00 + 1.78)	10.0 (3.27)	-0.2
	28.57 (25.00 + 3.57)	9.9 (3.26)	-0.3
4	25.00 (28.57–3.57)	10.1 (3.11)	0.3
	26.79 (28.57–1.78)	9.9 (3.09)	0.1
	**28.57 (4/0.14)**	**9.8 (2.99)**	**0.0**
	30.35 (28.57 + 1.78)	9.7 (3.11)	-0.1
	32.14 (28.57 + 3.57)	9.5 (3.08)	-0.3
4.5	28.57 (32.14–3.57)	9.6 (2.94)	0.0
	30.36 (32.14–1.78)	9.4 (2.99)	-0.2
	**32.14 (4.5/0.14)**	**9.6 (3.14)**	**0**
	33.92 (32.14 + 1.78)	9.4 (3.03)	-0.2
	35.71 (32.14 + 3.57)	9.1 (3.13)	-0.5
5	32.14 (35.71–3.57)	9.5 (2.96)	0.3
	33.93 (35.71–1.78)	9.3 (3.03)	0.1
	**35.71 (5/0.14)**	**9.2 (2.94)**	**0.0**
	37.49 (35.71 + 1.78)	9.2 (3.04)	0.0
	39.28 (35.71 + 3.57)	8.9 (2.92)	-0.3

Note: The bolded values represent the reference values

These results suggest that for children with GHD whose body weight falls within the range of fixed dose/0.14 ± 1.78 kg, the corresponding fixed dose yields comparable PK exposure, GV, and IGF-1 SDS levels. This supports the feasibility of a weight-banded dosing approach for GHD children, allowing them to be directly assigned a matching fixed dose.

## Discussion

The present study utilized a PopPK/PD model to develop and evaluate two optimized dosing strategies for Pegpesen in children with GHD. Our key findings demonstrate that, first, a proactive dose up-titration regimen, with escalations ranging from 12.3% to 26.0% every three months, can effectively mitigate the time-dependent waning of GV while maintaining IGF-1 levels within a safe range. Second, we established the feasibility of a simplified weight-banded dosing regimen, showing that for a given fixed dose, children within a weight range of ± 1.78 kg of the target weight achieve comparable pharmacokinetic exposure and pharmacodynamic outcomes without compromising efficacy. Together, these findings provide a model-informed framework for enhancing both the therapeutic efficiency and practical convenience of Pegpesen treatment.

Pegpesen is a LAGH designed for once-weekly administration, starting at a relatively low dose for the treatment of children with GHD. In a Phase 2/3 clinical study, once-weekly Pegpesen at 0.14 mg/kg/week demonstrated comparable efficacy and a similar safety profile, with lower immunogenicity, compared to daily rhGH at 0.035 mg/kg/day in prepubertal children with GHD [17, 29]. In a Phase 2 basket trial in prepubertal children with non-GHD conditions (i.e., ISS, SGA, or TS), once-weekly Pegpesen at 0.28 mg/kg/week showed similar efficacy and safety to daily rhGH at 0.067 mg/kg/day (Unpublished data). These findings confirm that Pegpesen has a favorable efficacy and safety profile within the 0.14–0.28 mg/kg/week dose range for children with short stature.

Based on observational data from Phase 1 trials in healthy subjects and Phase 2/3 trials in GHD treatment, a PopPK/PD model for Pegpesen in children with GHD was developed. Two optimized dosing regimens were simulated. The results confirmed that starting with a dose of 0.14 mg/kg/week and escalating every 3 months by 12.3%-26.0% can maintain higher GV levels in GHD children, addressing the gradual decline in GV over time. Additionally, the study validated that fixed doses (2, 2.5, 3, 3.5, 4, 4.5, 5 mg) are suitable for GHD children within a weight range of fixed dose/0.14 ± 1.78 kg, which improves medication convenience and reduces concerns about dosing errors for both doctors and parents.

Since its approval for pediatric GHD in 1985, the quest to optimize rhGH dosing strategies has been ongoing. In terms of administration frequency, rhGH therapy has evolved from three times weekly to daily, and now to once-weekly or even once-monthly administration with the advent of long-acting technologies. This evolution represents a dual optimization of convenience and efficacy, improving the quality of life for children requiring long-term treatment [1, 31]. Regarding dosage, the starting dose and subsequent adjustments for rhGH are primarily based on body weight or body surface area, as well as growth response [19]. Some researchers have also explored dose titration based on a target IGF-1 SDS value (e.g., 0 or + 2) to conserve dose and ensure safety [32]. Additionally, the initial treatment dose can be determined based on baseline characteristics of GHD children, such as annualized GV, pubertal stage, peak GH, and IGF-1 SDS levels [33]. Regardless of the strategy, the ultimate goal is to enhance treatment outcomes and help children with GHD achieve a better adult height. In reality, most explorations of optimized dosing have been driven by daily rhGH, for which the recommended dose range allows flexible starting doses and adjustment based on individual patient response. Unfortunately, despite the advancements in LAGH development, the exploration of varied dosing strategies has lagged behind that of daily rhGH. Current LAGH products typically employ a traditional approach where the total dose is calculated by multiplying body weight by a fixed unit dose. There is a clear need to address this gap to improve the therapeutic efficiency of LAGH for children with GHD.

First, a dose up-titration regimen was explored in this study. Previous studies of both daily rhGH and LAGH in children with GHD have consistently reported a gradual decline in GV starting from 3 months of treatment, with GV eventually approaching baseline levels after 3 or 4 years [24, 34]. The exact reasons for this waning effect are not fully understood but may be due to a gradual decrease in the body’s sensitivity to rhGH or a natural reduction in the patient’s intrinsic growth potential. Furthermore, a recent real-world study reported that the average treatment duration for Chinese children with GHD is only 1.69 years, with 11% of children discontinuing treatment due to perceived suboptimal efficacy [35]. These findings underscore the importance of developing optimized strategies to improve therapeutic outcomes. Based on the dose-dependent nature of GV, the PopPK/PD model in this study was used to simulate periodic dose escalations at different rates to identify a suitable regimen for maintaining GV at levels comparable to the early treatment phase. The dose adjustment frequency was set to every 3 months, consistent with the standard follow-up schedule for GHD patients [20]. The up-titration period (0.14–0.28 mg/kg/week) was designed to be completed within 18 months, reflecting the average treatment duration for children with GHD in China [35]. The results showed that the 12-month GVs for the 12.3%, 18.9%, and 26.0% up-titration groups were 9.51, 9.85, and 9.88 cm/year, respectively. However, at 24 months, the GV was 9.35 cm/year for all groups, indicating that while a faster dose escalation leads to a higher GV at 12 months, the effect of the dose increase reaches saturation by 24 months. This saturation effect raises a critical question for clinical practice regarding the optimal balance between efficacy and cost-effectiveness. Since different titration speeds led to the same two-year outcome in our model, it will be very valuable to explore which regimen is the most cost-effective in clinical research or practice based on these results. However, as these findings are based on a quantitative pharmacology model, prospective clinical trials are necessary to validate these strategies and determine the most appropriate regimen for clinical use. Indeed, the GV increase afforded by dose escalation is finite. The dose-response relationship for daily rhGH is well-established and characterized by a saturation effect. For instance, in children with GHD, first-year GV increases with dose up to approximately 0.05 mg/kg/day before reaching a plateau. Identifying this optimal dose ceiling is crucial for balancing efficacy with safety [36]. To our knowledge, however, a similar exploration to systematically define the dose-response curve and identify a potential saturation point for any LAGH in children with GHD has not yet been reported. Moreover, in studies of other LAGH, the attenuation in GV from the first to the second year of treatment has been reported to be as high as 20%-24%, posing a significant challenge for children in urgent need of height gain [24, 25, 37]. The significance of our up-titration approach also lies in mitigating this second-year GV decline. In the 12.3%, 18.9%, and 26.0% up-titration groups, the GV reduction from the first to the second year was only 1.7%-5.4%. This demonstrates that dose up-titration can effectively counteract the time-dependent decline in GV, allowing patients to maintain a robust GV over two years and thereby enhancing the overall efficiency of LAGH therapy.

It is well known that IGF-1 is the primary effector molecule of GH and a widely used safety biomarker [19, 38, 39]. Although there is no definitive evidence linking an upper limit of IGF-1 to GH treatment-related adverse events, the general consensus is that IGF-1 levels should be maintained below + 2 SDS in children with GHD [19]. In our study, the proportions of patients with IGF-1 SDS > + 2 in the first year were 4.5%, 7.5%, and 8.6% for the respective up-titration groups. In the second year, this proportion was 10.6% for all groups, which corresponds with the similar GVs observed. Compared to other LAGH studies, where the proportion of patients with IGF-1 SDS > + 2 ranges from 7.6% to 27.3%, the proportion in our study is considered acceptable [13–15]. Therefore, the three up-titration regimens proposed in our study are safe and manageable.

The second optimized strategy we explored was weight-banded dosing. Unquestionably, calculating the dose by multiplying a unit dose by body weight is the standard approach [1, 20]. With a constant unit dose, body weight becomes the sole determinant of the total dose. As children are constantly growing, their weight may change weekly, necessitating frequent dose recalculations. This increases the burden on physicians to update prescriptions and on caregivers to make frequent trips to the hospital, while also causing anxiety about potentially compromising efficacy if the dose is not adjusted promptly. A cross-sectional study on rhGH treatment adherence in GHD children identified forgetting injections or refills, being away from home, long-term injection fatigue, drug shortages, and inability to reach a pharmacy as significant barriers to adherence [40]. It is therefore crucial to reassure clinicians and families that minor weight fluctuations may not significantly impact overall efficacy, thus obviating the need for frequent dose changes. Based on the Phase 3 trial design and expected outcomes for Pegpesen in children with GHD, we simulated the appropriate body weight ranges for different strengths of Pegpesen (2–5 mg) under a 0.14 mg/kg/week regimen. Our simulations showed that the PK and IGF-1 profiles of the fixed dose/0.14 group and the fixed dose/0.14 ± 1.78 kg group were highly similar, suggesting comparable safety and efficacy. Therefore, under the 0.14 mg/kg/week regimen, each product strength of Pegpesen is suitable for children with a body weight within the range of fixed dose/0.14 ± 1.78 kg.

This study has some limitations. The findings are based on quantitative pharmacology modeling and are therefore theoretical in nature, requiring validation in clinical practice. We plan to conduct future studies to verify the feasibility of these optimized dosing strategies. Second, simulated annualized GVs tend to be lower than clinically observed values [17]. Therefore, the data presented in this study should be interpreted as indicative of trends and for comparing the relative differences between up-titration regimens, rather than as predictions of the actual GVs that will be achieved in clinical practice. Third, the model and its conclusions are specific to Pegpesen. Due to the diverse half-life extension technologies, unique PK/PD profiles, and distinct approved dosing regimens across different LAGH drugs, our findings should not be directly extrapolated to other LAGH therapies. We hope that the methodology and the concept of developing flexible, patient-centric dosing regimens can serve as a valuable reference for future research on other LAGHs, thereby contributing to the broader scientific understanding of this drug class.

## Conclusions

Based on a PopPK/PD model, this study for LAGH Pegpesen in GHD children proposes a dual strategy to optimize long-term therapy: a dose up-titration regimen to counteract declining GV while maintaining safety, and a simplified weight-banded dosing regimen to enhance treatment convenience without compromising efficacy.

## Supplementary Information

Below is the link to the electronic supplementary material.

Supplementary Material 1

## Data Availability

The datasets generated during and/or analyzed during the current study are not publicly available due to privacy reasons, but are available from the corresponding author on reasonable request.
