# Supplementary material for: Optimization of dosing regimens for the long-acting growth hormone pegpesen: A population PK/PD modeling approach
Source: J Endocrinol Invest. 2025 Nov 5;49(3):599–607. doi: 10.1007/s40618-025-02749-4 (PMC13018075; doi:10.1007/s40618-025-02749-4)
Supplement: Supplementary file 1 — Supplementary Material 1 [file 40618_2025_2749_MOESM1_ESM.docx]

**Optimization of Dosing Regimens for the Long-Acting Growth Hormone Pegpesen: A Population PK/PD Modeling Approach**

Youni Zhao^1#^, Fenfang Zou^1#^, Jianbo Gu^1^, Ruoyi He^1^, Yalin Yin^1*^

^1^ Xiamen Amoytop Biotech Co., Ltd, Xiamen, Fujian, P.R.China

^#^These authors have contributed equally to this work and share first authorship.

**^*^Corresponding author:**

Yalin Yin

Xiamen Amoytop Biotech Co., Ltd.,

Xiamen City, Fujian Province,

China

Email: [yinyalin@amoytop.com](mailto:yinyalin@amoytop.com)

**SUPPLEMENTARY MATERIALS**

**Figures**


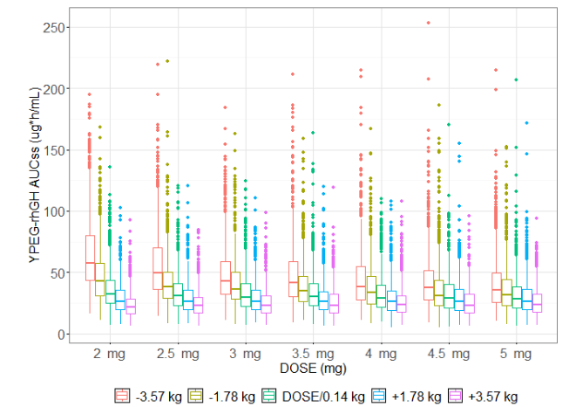


**Fig. S1** Steady-state pharmacokinetic area under the curve of Pegpesen by weight group

**Tables**

**Table S1** Baseline of the phase I study of Pegpesen in healthy adult subjects (NCT 01339182)

|  | **0.01 mg/kg/week (N=4)** | **0.03 mg/kg/week (N=8)** | **0.06 mg/kg/week (N=8)** | **0.12 mg/kg/week (N=8)** | **0.2 mg/kg/week (N=8)** |
| --- | --- | --- | --- | --- | --- |
| **Male, %** | 4 (100%) | 8 (100%) | 8 (100%) | 8 (100%) | 8 (100%) |
| **Age, years** | 35.3 (7.80) | 27.6 (2.56) | 26.6 (6.37) | 32.1 (8.56) | 34.5 (3.46) |
| **Height, cm** | 170.00 (8.49) | 166.88 (5.91) | 166.50 (7.60) | 170.63 (4.14) | 167.00 (6.19) |
| **Weight, kg** | 62.9 (5.46) | 62.7 (7.83) | 66.3 (6.73) | 65.0 (5.95) | 67.5 (4.15) |
| **BMI, kg/m²** | 21.8 (2.38) | 22.6 (2.77) | 23.9 (1.12) | 22.3 (2.07) | 24.2 (1.64) |

Data are mean (SD) unless otherwise stated. Abbreviations: BMI, body mass index.

**Table S2** Dose Correspondence between Pegpesen and Daily rhGH Based on Equivalent Steady-State IGF-1 Exposure over a One-Week Interval.

| **Daily rhGH Dose (IU/kg/day)** | **Steady-State IGF-1 Exposure (AUC) with Daily rhGH (AUEC ng*h/mL)** | **Pegpesen Dose (mg/kg/week)** | **Steady-State IGF-1 Exposure (AUC) with Pegpesen (AUEC ng*h/mL)** | **IGF-1 AUC Ratio (Pegpesen / daily rhGH)** |
| --- | --- | --- | --- | --- |
| 0.1 | 34571.62 | 0.14 | 33757.77 | **0.98** |
| 0.11 | 35218.05 | 0.15 | 35240.43 | **1.00** |
| 0.12 | 35810.6 | 0.17 | 37543.16 | **1.05** |
| 0.13 | 36373.69 | 0.18 | 38612.36 | **1.06** |
| 0.14 | 36877 | 0.2 | 40385.85 | **1.10** |
| 0.15 | 37342.96 | 0.21 | 41054.47 | **1.10** |
| 0.16 | 37780.55 | 0.22 | 41571.97 | **1.10** |
| 0.17 | 38185.43 | 0.24 | 42140.06 | **1.10** |
| 0.18 | 38533.57 | 0.25 | 42359.16 | **1.10** |
| 0.19 | 38851.74 | 0.27 | 42843.27 | **1.10** |
| 0.2 | 39155.86 | 0.28 | 43086.42 | **1.10** |

Abbreviations: rhGH, recombinant human growth hormone; IGF-1, insulin-like growth factor 1; AUC, area under the curve; AUEC, area under effect curve.
